# Evaluating Attitudes Towards Patient Care and Operations at Korle-Bu Outpatient Clinic

**DOI:** 10.5334/aogh.3073

**Published:** 2020-11-23

**Authors:** Lara J. Sokoloff, Benjamin Kornbluth, Lilly Taing, Adwoa Agyei-Nkansah, Stella Safo

**Affiliations:** 1 Icahn School of Medicine at Mount Sinai, New York, NY, US; 2 Korle-Bu Teaching Hospital, Accra, GH

## Abstract

**Background:**

Extensive research suggests that positive patient experience leads to improvement in patient health outcomes. Patient experience is particularly important in ambulatory care, where a patient builds a long-term relationship with a provider to manage his/her chronic illness over the span of years. Despite these known benefits, patient experience and its impact on health outcomes is poorly understood in low- and middle-income countries, where resources may be limited and primary care infrastructure spotty.

**Objectives:**

This paper aims to better characterize patient experience in a tertiary teaching hospital in Accra, Ghana.

**Methods:**

Forty qualitative interviews were conducted in the Outpatient Medical Clinic at Korle-Bu Teaching Hospital in Accra, Ghana. All interviews were transcribed and a qualitative analysis of central themes was evaluated by the study team.

**Findings:**

We found patients eager to share their views on clinical care in an ambulatory clinic in Ghana’s largest tertiary care center. Patients voiced desires for decreasing patient wait times, increasing wayfinding resources to navigate the clinic, creating appointment times, and implementing continuity of care with a single physician. The majority of patients also reported feeling actively engaged in their clinical care and emphasized their positive interpersonal interactions with providers.

**Conclusions:**

These findings suggest that patients described positive interpersonal experiences with providers at this ambulatory clinic, but identified numerous operational changes that could be made to vastly improve patient experience.

## Introduction

Patient experience covers the range of interactions patients have with all aspects of the healthcare system [1]. The quality of these interactions is influenced by a number of elements, including timely appointments, access to health information, as well as clear and concise communication between providers and patients [2]. Patients’ experience with their healthcare delivery has been observed to impact treatment outcomes; for example, patients who report better healthcare experiences often also report better treatment outcomes [3,4]. While much is understood about patient experience in high-income countries, there remains a relative paucity of research on how patients’ experiences impact clinical care in low- and middle-income settings. This paper explores patients’ experience with their ambulatory clinical care in a major tertiary care center in Ghana, West Africa.

The lack of patient experience research in low- and middle-income countries may be explained by several factors. Some studies suggest that a subset of patients in sub-Saharan Africa may have low health literacy levels, which may limit understanding of both the healthcare system and of individual health directives, ultimately compromising a patient’s ability to adequately steward his/her health maintenance [5]. For example, Roder-DeWan et al. 2019 found that when presented with multiple vignettes describing poor quality healthcare—both poor interpersonal and technical skills—survey respondents from 12 lower/middle income countries overwhelmingly reported the care as good, very good, or excellent [6]. The findings caution that patient satisfaction surveys may bias upwards given patients’ low-quality expectations. In contrast, another body of research suggests that some patients in Sub-Saharan Africa demonstrate impressive health-seeking behavior and are active agents in their healthcare [7,8]. Together, this research suggests that patients strive to make active choices in their healthcare, but the basis on which these decisions are being made, and whether those decisions are based on informed healthcare knowledge, remains poorly understood.

Ghana represents an ideal context to evaluate the nature of patient experience in a developing landscape because of its growing investment in managing chronic illnesses. Ghana’s burden of illness has shifted from infectious diseases such as malaria and tuberculosis to non-communicable, chronic diseases (NCDs). Both hypertension and diabetes are at historical highs in Ghana, with an estimated prevalence of 36.6% and 8.3% respectively in adults 18 and over [9]. To help manage these chronic illnesses, the country has made significant investments in primary care delivery with the 2003 National Health Insurance Scheme (NHIS), which seeks to provide universal health coverage to Ghanaians by removing financial barriers to accessing care. Despite challenges to implementation, the NHIS has become a model for neighboring countries in the region [5]. In recent years, the focus on chronic care and increased access to healthcare services via the NHIS makes Ghana a good case study to evaluate patient experience in a developing context.

A review of research on patient experience in Ghana suggests a mixed picture. Ofei-Doodo’s 2019 analysis of WHO survey data on over 2,500 Ghanaian patients found that 90% of patients were satisfied or very satisfied with their care, and that patients who reported higher satisfaction with their care also reported better treatment outcomes [10]. In contrast, data published in 2007 from Ghana’s Ministry of Health suggests that both patients and providers viewed care as not reaching levels of expectations [11]. More recently, studies by Essiam in 2013, as well as Alhassan et al. in 2015, reported patients were dissatisfied with their care, in spite of their endorsement of excellent technical quality of the healthcare they received [12,13]. Therefore, there is a mixed picture around patient satisfaction in Ghana: while some evidence exists pointing towards satisfaction with care, other studies suggest that there are areas of opportunity for providers to improve patient care. An evaluation of patient experience in Ghana also offers an opportunity to better understand some of the drivers of patients’ experience. These include the technical skill of the provider, internal relations between the patient and health care staff, and the physical domain in which the patient receives care. Furthermore, much of the existing research on patient experience in developing countries has focused predominantly on patients’ experiences in rural settings [14,15]. To our knowledge, minimal research has been conducted in major tertiary care centers.

In light of the variability of research on patients’ experiences in low- and middle-income countries, our study assessed patient experience at Korle-Bu Teaching Hospital in Accra, the largest teaching hospital in Ghana. We chose the patient population at Korle-Bu because we believe that patients accessing care there are uniquely motivated in managing their health, since many of them travel great distances and incur great cost to obtain care at Korle-Bu. To evaluate these contributors to patient experience in Ghana, we conducted 40 qualitative interviews with both patients and caregivers over the span of five weeks.

## Methods

### Study Site

The study was conducted in the Outpatient Medicine Department (OPD) of Korle-Bu Teaching Hospital in Accra, Ghana from June to July 2018. Korle-Bu is one of only four teaching hospitals in Ghana and is the country’s largest tertiary care center [16]. The hospital currently employs roughly 25% of all qualified physicians in Ghana, including a number of Ghana’s most highly specialized physicians [17]. Korle-Bu is a 2,000-bed facility with 250 daily admissions and 1,500 daily outpatient visits. It is the nation’s leader in specialty care with access to advanced laboratory and imaging offerings and healthcare technology [18].

### Patient Population and Recruitment

At Korle-Bu, all-comers (both new and returning patients) to the OPD were provided the opportunity to enroll in the study. The OPD head nurse made a daily announcement informing patients in the waiting room of the research study. Participation was voluntary and no incentives were offered. Patients or caregivers were required to be 18 years of age or older and speak English, Ga, or Twi. Patients were excluded if they appeared acutely ill or had unstable vitals, although no patients in these categories volunteered for the study. Forty eligible patients and/or caregivers were interviewed over the course of five weeks by study staff. Patients were interviewed after they had their vitals obtained by the nursing staff before their visit with the clinician.

### Study Questionnaire and Interviews

An interview questionnaire was created by the study staff based on insights from literature on patient experience and engagement. The interview guide included open-ended questions on patients’ satisfaction with the clinic flow and infrastructure and with providers, as well as on patients’ suggestions for improvements to the clinic. Interviews were conducted in a private room adjacent to the main clinic waiting room. Both primary researchers (BK, LS) and a Ghanaian interpreter who spoke English, Twi, and Ga were present for all 40 interviews. Prior to initiating each interview, signed informed consent was obtained from all study participants. Each participant received a printed consent and was given time to read through the form. Consent forms were then read aloud in English by the primary researchers, and when necessary, translated into Twi or Ga by the qualified interpreter. The primary researchers then encouraged participants to ask any additional questions. Full understanding of the consent was confirmed and participants were invited by the primary researchers to sign the consent form. Interviews were audio recorded with permission from participants. All transcripts were transcribed the day of the interview.

### Analysis

Each transcribed interview was reviewed by both primary researchers and general themes were recorded. An inductive thematic approach was utilized to generate a coding framework and create coding headers, as well as sub-codes [18]. The finalized codes were used to evaluate all 40 interviews within the QSR International’s Nvivo 12 software. Each interview was coded by the two primary researchers in addition to a third researcher with no prior affiliation to the study (LT). The team then examined the frequencies and relationships of codes to identify broad themes within the interviews. All study procedures were approved by the Institutional Review Boards of both the Icahn School of Medicine at Mount Sinai and the Korle-Bu Teaching Hospital (IRB-18-00458).

## Results

### Patient Demographics

Forty patients and caregivers in the Korle-Bu Teaching Hospital OPD (Table 1) were interviewed over the course of five weeks; twenty-seven (67.5%) were patients and 13 (32.5%) were caregivers. Participant ages ranged from 27 to 80 (mean = 50.1). Most participants (34 participants, 85%) were returning to Korle-Bu, while 6 (15%) were new patients or caregivers of new patients. Thirty-one (77.5%) participants were from the Greater Accra region and the remaining were from the Central Region (7; 17.5%), Western Region (1, 2.5%), or Eastern Region (1, 2.5%). Time spent traveling to Korle-Bu varied among participants with a range of <1 hour to upwards of 9 hours, as follows: ≤1 hour (15, 37.5%), 1.5 hours–2.5 hours (12, 30%), >3 hours (6, 15%).

**Table 1:** Demographics.

Variable	Participants *(n%)*

**Baseline Demographics**	
Age < 39	11 (28.2%)
Age 40–55	14 (35.8%)
Age 56–70	10 (25.6%)
Age > 71	4 (10.2%)
Age Range	27–80
Patient	27 (67.5%)
Caregiver	13 (32.5%)
First visit to KBTH	6 (15%)
Returning visit to KBTH	34 (85%)
**Regional Demographics**	
Accra	31 (77.5%)
Central	7 (17.5%)
Western	1 (2.5%)
Eastern	1 (2.5%)

### Major Themes

Two central interrelated themes emerged from our interviews on factors impacting patient experience at Korle-Bu’s outpatient clinic. First, participants identified multiple operational barriers to obtaining medical care and spontaneously offered a number of suggestions for improvement. Second, most participants endorsed feeling positively about their interpersonal experiences and engagement with clinic staff and cited the high-quality physicians as their reason for seeking care at Korle-Bu. Yet some participants described feeling uncomfortable with speaking to their clinicians about all aspects of their medical care, suggesting limitations to the closeness in their interpersonal relationship with providers.

### Theme: Operational Barriers to Care

Participants listed a number of operational challenges with seeking care at Korle-Bu and suggested improvement activities that the clinic can undertake to address these barriers to care.

### Barrier 1: Challenges with Navigating the Clinic

**Figure 1:**
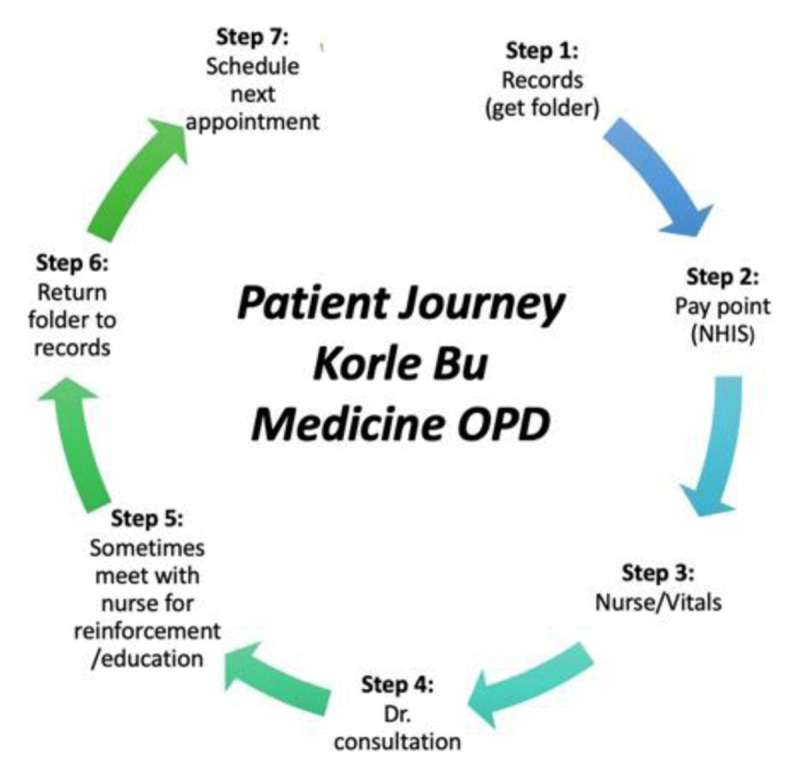
Patient Journey Korle-Bu Medicine OPD.

A majority of participants discussed significant operational barriers that made the clinic difficult to navigate. Participants described the clinic flow as “clandestine” and “cumbersome.” A patient’s journey during a single clinic visit includes checking in at the front desk to retrieve their folder, finding and then waiting in line to register with the National Health Insurance Schema to pay, finding a place to wait for the nursing staff who will obtain vital signs, and finally, waiting for the visit with the physician (Figure 1). As one patient explained, “The procedure is not straightforward here—they (clinic staff) say go here, come here, come and sit here, and go and sit outside there. No one knows where to sit” (Interview 1). As a result, most participants reported relying on nursing staff to help navigate the clinic. At their first visit in particular, some participants reported that they found the OPD impossible to navigate without the assistance of staff. “I think it’s complex if you don’t chance upon one of the nurses out there” (Interview 32).

### Suggestion 1: Institute Directional Aids in Clinic

As a result of the expressed way-finding difficulties, a number of participants recommended instituting directional aids to help streamline the clinic flow. As one participant expressed: “I think we should have something when you enter that says where to go, something like a flow chart. This is an old ward and it’s all messed up. People get confused and don’t know where to go” (Interview 4). Because some patients and caregivers coming to Korle-Bu may not be literate, participants also suggested that directional signs be illustrated as arrows and figures, and be aesthetically pleasing and prominent enough to draw attention. “One very important thing is that there are directionals for those who can’t read. Take your card here, pay NHIS here, pick this up from here, do this. Once you arrive, are you new to Korle-Bu? Maybe I think it will help, it will help!” (Interview 32).

### Barrier 2: Long Wait Times Tied to a Physician Shortage

Many participants expressed concern with the shortage of the clinician workforce and long wait times. One participant explained: “You come here in the morning and… leave here at 4:00pm in the evening. How come I sit in here the whole day? In Ghana, you go to the hospital… [and if it’s a] government hospital, you will spend the whole day” (Interview 6). Participants explained that the long wait times are in part due to how they are scheduled. Patients are provided a clinic day to come in for a visit and are seen on a first come first serve basis. As a result, most participants we spoke with described arriving very early in the morning to be the first to be seen when the clinic opened. Not surprisingly, lines are long at each of the points of care, and patients often spend upwards of five hours waiting to be seen. Participants also noted the impact of not having enough physicians as part of the delayed clinic flow: “[Waiting] is due to lack of adequate doctors. The doctors are few, the patients are many. It makes the doctor get exhausted in no time. One doctor has to see about 500 patients a day, it’s just too much” (Interview 26). Finally, a few participants noted that physicians arrive late to clinic, sometimes not until mid-day, which also contributes to delays.

### Suggestion 2: Increase the Clinician Workforce and Institute Appointments to Reduce Wait Times

To address the long wait times, almost half the participants suggested training and hiring more doctors, which they believed would help significantly reduce their time spent waiting for care. As one person explained: “Maybe if they had enough doctors, or so-called ‘big doctors,’ around, it will be easier. If there’s only one ‘big doctor’ and all of us have to see the same ‘big doctor,’ it will be a lot for one person to handle. But if there are more than one, two, or three ‘big doctors,’ then it will be easier” (Interview 21). We interpreted the term “big doctor” here to be the attending physicians who manages trainee clinicians. This participant was noting the lack of physicians in general, and attending physicians in particular, as a major contributor to prolonged clinic times. Participants also characterized the physician shortage as a systemic issue “that depends on the government” (Interview 26). This participant explains this belief as follows: “[The clinician shortage] starts from the training of the doctors. If the government can come in and train potential doctors, they would not have this ratio problem of one doctor to about 500 patients. The government should train people who have the passion to become doctors” (Interview 26). While addressing the clinician shortage is a long-term solution to reducing wait times, implementing appointment times may have a much more immediate impact. As one participant explains: “Not having appointments needs to change, we need to give appointments and follow it to the letter, then I don’t have to wait so long” (Interview 3). Another participant used herself as an example noting how appointments would streamline the whole process, reduce stress, and allow her and others to not have to choose between missing an entire day of work and going to the doctor. “Like me, if I’m going to work, I can book, say at 2:00. I’ll just come, maybe 1:30 I’ll be here, do the photocopy, by 2:00 I’m ready. Then 30 minutes, one hour, I’m off. Instead of sitting here, [with] my pressure going up” (Interview 8).

### Barrier 3: Interruption in Continuity of Care

Multiple participants commented on their lack of follow-up with the same clinician. They expressed frustration at spending time retelling their clinical histories, as well as having to get acquainted and comfortable with new physicians who have different practicing styles and personalities. One participant explained, “I realize that the first time you come, you see a specialist. But the next time you come, you are given to a new doctor who is available. I feel that is a bit worrying because the first one you see, you tell everything. Then the next time you come with another doctor, sometimes he has to read everything, and maybe his style is not as the first one, so you feel a bit like something is missing” (Interview 8). In addition to what this participant describes as a lack of comfort with the clinical style of care delivery from multiple providers, other participants noted that lack of continuity can lead to medication changes and unnecessary lab or imaging testing. For example: “It is better that you have a personal doctor who knows your history, who follows your history… When this [other doctor] comes, [the new doctor] asks you [to] go for another lab, you come, you go and meet another [doctor], and you have to go and do this lab again—it’s stressful. But if you have one doctor who will be attending to you, who knows your problem, he knows how to at least help you out, then that is the best. Changing doctors is not the best” (Interview 16).

### Suggestion 3: Implement Continuity of Care with Providers

Many participants expressed interest in seeing the same physician each time they come to clinic. Participants felt strongly that seeing the same provider regularly allowed the patient and clinician to become more familiar with each other, “Because if I have to see the same [doctor], I know there has been a rapport, a relationship committed. And if you have a situation where patients can be assigned to specific doctors, it would be very easy, because the doctor has a history of the patient” (Interview 32). These lasting relationships could increase efficiency and streamline care. As one patient reasons: “If you have one doctor who you see every time, he knows where he started so he knows where he’s supposed to continue. That saves time” (Interview 25). As a suggestion for improvement, one participant explained how his provider advised him to approach future visits: “So the doctor told me, if I come, I should tell them [front desk and nursing staff] that I have a specific doctor… So every day I ask for my [same] doctor” (Interview 30).

### Theme: Interpersonal Experiences with Staff

Interpersonal relationships with the clinical staff and physicians represented a second major theme that had a significant role in patients’ experience and overall satisfaction.

### Positive Experiences with Staff and High Patient Engagement

Patients were overwhelmingly satisfied with the quality of their clinical encounters, with over two thirds of patients interviewed providing positive feedback. Many participants noted the kindness of the clinical staff and physicians. For example: “The doctors are friendly and they make sure they check you well. They talk with you as if you are equals. They want to know your problem and help you” (Interview 26). Other participants highlighted positive interactions with the nursing staff: “The care, the way [the nurses] receive you, and the way they care for you, how they feel when you are sick and you come here, the nurses are very, very good” (Interview 30). Similarly, another patient noted: “The nurses are good and accommodating. If you don’t know something, they can give you some schooling” (Interview 10). Patients’ reflections of the physicians and nurses as “accommodating”, “good”, and “friendly” demonstrate their overall positive assessment of the interpersonal care at Korle-Bu.

It is not surprising then that these descriptions of positive interpersonal interactions led to patients expressing feeling engaged with their care. We evaluated engagement along a number of parameters, each of which were endorsed by a minimum of 50% of participants (Table 2). In particular, 36 of 40 participants reported feeling confident asking their clinicians questions regarding their medical concerns. “Some Africans feel shy to ask questions, but for me I don’t feel shy. I encourage asking [questions] to educate [myself]” (Interview 18). Multiple participants also noted that the language used by providers improved the quality of the visit. One patient described the visit itself having a healing effect: “I feel that I’m even healed already after talking to the doctor. Sometimes, it’s not medicine, it’s the way you talk to the doctor, the way he answers everything, then you will be healed” (Interview 30).

**Table 2:** Positive Clinical Experiences.

Positive Clinical Experience: Patient Experience	N = 40 *(n%)*

Comprehends medical plan	31 (77.5%)
Confident carrying out medical plan	23 (57.5%)
Confident asking questions	36 (90%)
Confident expressing medical concerns	20 (50%)
Reassured by care	32 (80%)
Felt listened to	35 (87.5%)
Understands provider clearly	32 (80%)

In addition to endorsing strong interpersonal relationships with their clinicians, many participants also reported holding Korle-Bu itself in high esteem (Table 3). Participants cited the hospital’s high-quality medical care as their primary reason for seeking services there, despite the great distances many traveled. Participants explained that as the country’s largest hospital, Korle-Bu employs the most specialists and has the most available specialized resources; some also described Korle-Bu as the “last stop” and a place to which they are consistently referred to from other health facilities. Several participants expressed a strong belief that the physicians at Korle-Bu are some of the most highly skilled in all of Ghana: “[Korle-Bu is] the biggest hospital, you can’t get these doctors, consultants, nurses in other places” (Interview 1). Another participant added: “I’m sure they [Korle-Bu providers] actually saved my life. The best doctors in the country are here. That’s why I keep coming back here” (Interview 17). Table 3 provides a snapshot of the myriad reasons for which patients sought care at Korle-Bu.

**Table 3:** Patient Explanations for Seeking Care at Korle-Bu.

Reasons for choosing/returning to Korle-Bu	Participant Quotes

Large size of institution and advanced technology	“It’s a big institution so you can be transferred to other departments and subspecialists throughout the hospital.” (Interview 5)
	“She [the patient] believes, so far, this is the biggest hospital we have in where she can get qualified doctors to take care of her. Before we got here, everyone was advising us to not go to any herbalists. She insisted that we should come here [Korle-Bu] first. And she hasn’t seen the herbalist since.” (Interview 21, patient caretaker)
Top physicians and availability of specialists	“I love their service. When it comes to West Africa and Nigeria, [Korle-Bu] gives adequate healthcare.” (Interview 18)
	“Oh yeah, the best doctors in the country are here. That’s why I keep coming back here.” (Interview 17)
	“There are good Doctors and specialists and if you are lucky enough to meet them its good because they treat you well and by God’s grace you will be well.” (Interview 7)
Positive patient outcomes	“She [the patient] says if the doctor will take good care of her, she will always come.” (Interview 31, patient caretaker)
	“I like the way the service is. They diagnose you and find out what is wrong and take care of you.” (Interview 27)
Korle Bu as “last stop”	“If I cannot [obtain care] at Korle Bu, then I should close my coffin.” (Interview 5)
	“Wherever you go, even private hospitals, you always end up being referred back here [to Korle-Bu] anyway.” (Interview 14)
	“Here [Korle-Bu] is the last stop, if they can’t help you here then you are going to be dead.” (Interview 5)

Despite strong approval of Korle-Bu by many study participants, the next section demonstrates that many participants struggled to effectively communicate with their providers; the reason for this inadequate or incomplete communication ranged from believing some topics to be outside of the patient-doctor boundaries, to being concerned with how raising certain topics would impact the delivery of care.

### Participants’ Challenges with Provider Communication

As described, participants overwhelmingly reported feeling positive about and comfortable with their clinicians and with the care provided at Korle-Bu. However, participants described two instances—the discussion of non-medical problems and the approach to disagreeing with their clinicians—where they felt challenged with fully expressing their opinions.

Twenty-two of our 40 interviewed participants stated they would be hesitant to discuss “non-medical” issues with their physicians. These “non-medical” issues such as financial challenges, unstable housing, or limited access to food often impact patients’ health outcomes, such as complicating medication adherence or preventing adequate rest and recuperation. Participants explained that they chose not to share non-health information for three primary reasons. First, participants’ physicians did not directly ask: “[The doctors] don’t make room for that. They don’t ask you what you are also doing to keep yourself healthy and strong. If they do, I would tell them, but they don’t. So, I keep that to myself” (Interview 17). Second, participants did not believe it was within the physician’s area of expertise: “I wouldn’t have been able to feel comfortable, because I would be thinking that is not [the doctor’s] area of operation. If I have a family problem, I talk to a counselor; if I have a financial problem, I have to talk to a banker or something. So, I wouldn’t feel comfortable talking to the doctor” (Interview 32). Finally, some participants perceived that their physician would not be able to help: “Personal issues, I’m not too sure I can discuss that with the doctors. It’s not the problem of the doctor if you have problems with money or anything around here. If you have problems with money, you have to go look for your own money and take care of yourself. The doctor can do nothing about that” (Interview 21).

In addition to the reluctance to communicate certain “non-medical” concerns to the physician, our interviews uncovered a common theme around healthcare autonomy and deference to clinician. Twenty-five percent of participants described deferring to their physicians’ medical opinions, as they believe their physicians are the authority on matters of health, and not doing so would be a waste of time and effort: “If I don’t go through with it [the physician’s medical plan], then why should I report here [e.g. come to the clinic]? So, I have to take the instructions given to me, and any other advice that he gives me. Whatever the doctor asked me to do with regards to my health, I have to do” (Interview 16). Another participant explained: “He is the doctor taking care of me. So, I have to listen to him and do what he says” (Interview 37).

## Discussion

Patient participants at the Korle-Bu Outpatient Clinic in Accra, Ghana identified a number of operational barriers to obtaining care, including poor clinic organization, long wait times, and lack of continuity of care with their providers. Despite these challenges, participants also endorsed overall positive patient experiences with clinic staff and providers. Participants offered many suggestions for improvement, which included adding directional signs in the clinic to improve clinic flow, training and employing more physicians to provide care, creating patient appointment times, and instituting continuity of care with a singular physician.

Wait times and concerns about lack of continuity of care—both primarily driven by a physician shortage—presented some of the most concerning operational barriers participants highlighted in this study. As of 2017, Ghana only has 0.10 physicians per 1,000 peoples; by comparison, the United Kingdom has 2.8 physicians per 1,000 people, while the United States has 2.6, a roughly 15-fold difference [19]. This places Ghana at 14^th^ overall in terms of physician to patient ratio on the African continent, and fourth in the West African sub-region. However, we recognize there are significant operational barriers and costs to increasing the physician workforce in Ghana, and recommend improvements that may be taken while the physician workforce shortage is addressed. For example, participants in our study suggested reorganizing clinic days, instituting appointment times, and ensuring continuity of care with a single physician as improvements to help alleviate the long waits.

Despite some of the reported obstacles to seamless care, our study found that patients reported having very positive interpersonal interactions with their providers. Patients felt that they could trust the advice from their providers and that they were being cared for by some of the best medical providers in the country. Interestingly, some participants concurrently reported rarely sharing non-medical challenges (such as lack of finances to fund care) that could negatively impact their health outcomes; this subset of patients also noted that they deferred their medical decision making to their physicians. This apparent disconnect between the lack of free autonomous speech with providers and our participants’ report of excellent clinical care may be explained by Lee and Lin’s research, which found that patients have varying preferences in involvement in their care. Some patients may want active roles in their care decisions, while others want to rely entirely on physicians to make those decisions [20]. This differential expectation for clinical care and healthcare autonomy may account for why our study’s participants reported high provider satisfaction alongside restricted communication and healthcare decision-making.

Similarly, participants reported operational challenges while highlighting excellent provider care. Participants in this study appeared to separate their experience with the physical clinic environment from their interpersonal interactions with providers. This parallels Chang’s et al. findings that patients consider these realms—operational and interpersonal—separately [21]. Despite this discordance, research still suggests that operational barriers can in fact undermine the care patients ultimately receive [15]. More specifically, Amanakwah et al. found that organized clinical environments reduce stress, promote healing, and provide patients opportunities to express themselves openly with providers, ultimately leading to higher patient satisfaction [14,22]. Innovative changes that improve clinic flow and promote a conducive environment for care are likely to increase patient satisfaction with a healthcare facility and also improve provider interactions and overall healthcare outcomes [23,24,25].

This research contributes to a growing body of work on patient experience in Ghana. In particular, our study offers a unique qualitative lens that allowed patients to more holistically assess and discuss the care they received. Through the use of semi-structured interviews, patients had the opportunity to elaborate on their care experience, offering targeted feedback for their providers and the clinic as a whole. We believe this methodology allowed us to better understand patients’ baseline health literacy in addition to their satisfaction with care, while also exploring complex dynamics within aspects of patient satisfaction. In focusing on an urban setting, our study shifted away from rural settings which predominate patient experience research in developing nations, instead focusing on a major tertiary care center in a large urban hub.

Our study had a number of limitations. We conducted this study in a single hospital in Ghana, impacting the generalizability of our results, as this clinical setting may not reflect the setting in which many Ghanaians, especially those in non-urban environments, may receive care. This study was conducted over the span of five weeks during the summer months, and it may not be as representative of patients who come to the clinic throughout the rest of the calendar year. In addition, this study was managed by foreigners and interviews were conducted via interpreters: this cultural difference may have influenced participants level of openness in their replies. Finally, with any study conducted within the clinical sphere where patients are seeking care, patients may be reluctant to fully express their concerns or issues.

## Conclusion

Our study findings are important for hospital staff, clinicians, and administrators, who may use the focused feedback presented in this study to facilitate operational changes that seek to increase patient satisfaction and improve health outcomes. Our findings suggest that while some immediate steps can be taken, such as improving signage and clinic flow, there are also opportunities to implement larger scale modifications, such as setting appointment times, providing continuity of care with a single clinician, and the hiring of more physicians to provide care. Future research should investigate institutional hurdles to implementing patients’ suggestions.
